# Study protocol: a multi-professional team intervention of physical activity referrals in primary care patients with cardiovascular risk factors—the Dalby lifestyle intervention cohort (DALICO) study

**DOI:** 10.1186/1472-6963-12-173

**Published:** 2012-06-22

**Authors:** Emelie Stenman, Matti E Leijon, Susanna Calling, Christina Bergmark, Daniel Arvidsson, Ulf-G Gerdtham, Kristina Sundquist, Rickard Ekesbo

**Affiliations:** 1 Center for Primary Health Care Research, Lund University, Malmö, Sweden; 2 Bara Health Care Centre, Skåne County Council, Skåne, Sweden; 3 Dalby Health Care Centre, Skåne County Council, Skåne, Sweden; 4 Department of Economics, Lund University, Malmö, Sweden; 5 Health Economics & Management, Institute of Economic Research, Lund University, Malmö, Sweden

## Abstract

**Background:**

The present study protocol describes the trial design of a primary care intervention cohort study, which examines whether an extended, multi-professional physical activity referral (PAR) intervention is more effective in enhancing and maintaining self-reported physical activity than physical activity prescription in usual care. The study targets patients with newly diagnosed hypertension and/or type 2 diabetes. Secondary outcomes include: need of pharmacological therapy; blood pressure/plasma glucose; physical fitness and anthropometric variables; mental health; health related quality of life; and cost-effectiveness.

**Methods/Design:**

The study is designed as a long-term intervention. Three primary care centres are involved in the study, each constituting one of three treatment groups: 1) Intervention group (IG): multi-professional team intervention with PAR, 2) Control group A (CA): physical activity prescription in usual care and 3) Control group B: treatment as usual (retrospective data collection). The intervention is based on self-determination theory and follows the principles of motivational interviewing. The primary outcome, physical activity, is measured with the International Physical Activity Questionnaire (IPAQ) and expressed as metabolic equivalent of task (MET)-minutes per week. Physical fitness is estimated with the 6-minute walk test in IG only. Variables such as health behaviours; health-related quality of life; motivation to change; mental health; demographics and socioeconomic characteristics are assessed with an electronic study questionnaire that submits all data to a patient database, which automatically provides feed-back to the health-care providers on the patients’ health status. Cost-effectiveness of the intervention is evaluated continuously and the intermediate outcomes of the intervention are extrapolated by economic modelling.

**Discussions:**

By helping patients to overcome practical, social and cultural obstacles and increase their internal motivation for physical activity we aim to improve their physical health in a long-term perspective. The targeted patients belong to a patient category that is supposed to benefit from increased physical activity in terms of improved physiological values, mental status and quality of life, decreased risk of complications and maybe a decreased need of medication.

## Background

The present protocol describes the trial design of a Swedish primary care intervention cohort study, which examines whether an extended, multi-professional physical activity referral (PAR) intervention is more effective in enhancing and maintaining the level of physical activity than physical activity prescription in usual care. The target group is patients with newly diagnosed type 2 diabetes and/or hypertension. The study is designed as a long-term intervention with recurrent analyses of self-reported physical activity levels as well as the effects of physical activity on health-related variables and cost-effectiveness.

Hypertension and type 2 diabetes belong to the major risk factors for cardiovascular disease that can be modified by a change in lifestyle, such as increased physical activity [1-3]. Several studies confirm that physical activity reduces the systolic and diastolic blood pressure in patients with hypertension, and improves the glycemic control in subjects with type 2 diabetes [2,4]. The evidence suggests that lifestyle modifications and exercise therapy are as effective as pharmacological treatment in selected cases. According to current guidelines, lifestyle modifications should be the first-line treatment alternative for patients with diagnose, or risk for, type 2 diabetes or hypertension [5,6]. Daily or almost daily aerobic physical activity of moderate intensity, such as brisk walking or swimming, for at least 30 minutes is recommended for both conditions [5-7].

### The extended PAR intervention

Written prescriptions of physical activity — in Sweden commonly referred to as PARs — provide an attractive alternative for encouraging patients to increase their physical activity levels and thereby improve health and quality of life. These prescriptions are provided by registered health care professionals in primary care and hospitals [8,9]. The effectiveness of Physical activity or exercise referrals and exercise referral schemes show mixed results. A review article shows that physical activity referrals and exercise referrals increase the physical activity levels in certain populations, although the effect tends to wear off over time [10]. A more recent review state that uncertainty remains about the effectiveness of exercise referral schemes for increasing physical activity and improving health [11]. The Swedish Council on Technology Assessment in Health Care, SBU, state that advice and counseling of patients increases physical activity by 12–50% for at least 6 months, and that a supplementing prescription can increase the activity even further [12]. Clearly, more research is needed to elucidate predictors of successful adherence to this kind of prescriptions or referrals.

Factors that influence the level of physical activity are often classified as individual (e.g. demography, cognitive skills, self-efficacy), interpersonal (e.g. encouragement from family, friends or care givers) or environmental (e.g. culture, physical surroundings, access). Self-efficacy, self-perceived health status and social support belong to the most important predictors of exercise behavior [13]. Other reasons for performing physical activity include improved appearance, enjoyment of the activity itself, social interaction and stress relief [13,14].

To optimize the adherence to PARs and increase the level of physical activity in patients with newly diagnosed type 2 diabetes or hypertension, we will introduce an extended PAR intervention. The aim is to give the patients sufficient support to become regularly active and prevent relapse into inactivity.

The step-based intervention involves a multi-disciplinary primary care team, which may include e.g. physician, hypertension/diabetes nurse, psychologist, physiotherapist or occupational therapist. The team will, in consultation with the patient, customize a PAR based on the summarized results of a pre-test, which includes a computerized study questionnaire, a 6-minute walk test, and physiological status. The study questionnaire is designed to capture factors that may impact the level of physical activity such as self-efficacy and general health behaviors [15]. The multi-disciplinary team will use the principles of Self-Determination Theory (SDT) and Motivational Interviewing (MI) throughout the patient meetings to improve the adherence to the intervention.

The suggested PAR concept is somewhat different from the original Swedish PAR schemes, which are written prescriptions of individual activities, such as jogging, or group-based activities, such as aerobics, and commonly designed according to the national recommendations for respective disease. In our extended concept, the PAR is based on the patient’s preferences and interests, physical and mental status, and motivation.

### Self-Determination Theory (SDT)

**Figure 1:**
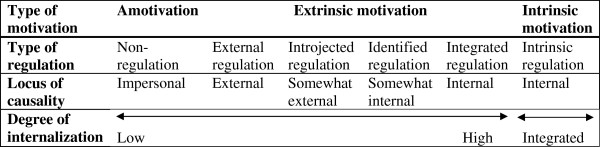
**The Self-determination continuum.** The figure shows the different types of motivations, regulations and loci of causality according to SDT. One of the aims is to evaluate the patients’ degree of internalization at baseline and changes in along the continuum during the course of the study. Adapted from [17,18].

SDT is a theory of motivation, which was initially developed by Deci and Ryan [16,17]. SDT focuses on internalization of motivation in order to increase qualities such as perseverance [18], which is crucial for maintenance of profound lifestyle changes. SDT defines 3 innate psychological needs that are crucial for internalization of motivation: *autonomy*, *competence* and *relatedness*[17,18]. Satisfaction of these 3 needs improves the internalization of motivation along a continuous scale that extends from *amotivation* (lack of intention to act), over *extrinsic motivation* (when the activity is performed to achieve outcomes that are separable from the behaviour itself), to *intrinsic motivation* (which comes from the satisfactions of the behaviour itself) [16,17] (Figure 1). As can be seen in Figure 1, extrinsic motivation is divided into 4 subgroups: *external regulation*, *introjected regulation*, *identified regulation* and *integrated regulation*[16,18]. Thus, the patients’ motivation can move stepwise within extrinsic motivation.

### Motivational Interviewing (MI)

**Figure 2:**
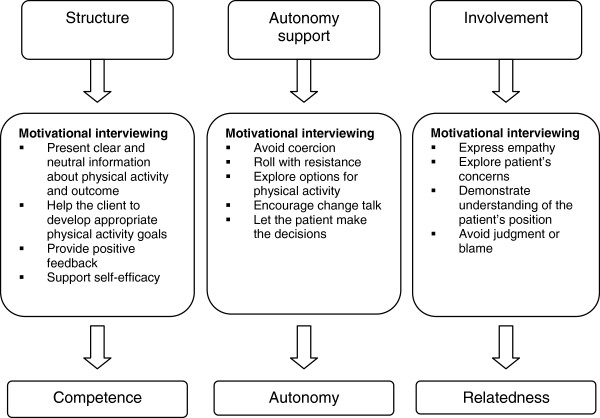
**Correlation between Self-determination theory and Motivational Interviewing.** Adapted for PAR intervention from [19].

The 3 innate psychological needs *competence*, *autonomy*, and *relatedness*, defined by SDT, are believed to be promoted by 3 environmental factors: *structure, autonomy support and involvement*[19]. These 3 environmental factors can in turn be supported by MI. MI is a client-centred counselling style that aims to elicit behaviour changes by helping clients to explore and resolve ambivalence [19,20] (Figure 2). The general principles of MI have been shown to correspond very well with the intentions of motivational internalization in SDT: whereas SDT provides a theoretical framework for internalization of motivation, MI is more practically oriented and can help to translate the SDT concept into practice [19,21].

The principles of MI have previously been shown to be effective in improving health behaviours such as diet, exercise and addiction treatment [21].

### Health economic aspects

One of the main priorities of Sweden’s National Public Health Policy is to increase the level of physical activity in the population and a systematic literature review published by the Swedish Council on Technology Assessment in Health Care points to health economic evaluations of physical activity intervention as one area of special importance in future research. This taken together with the relatively scarce, and of varying quality, health economic studies on interventions promoting physical activity that exists today, makes this field of special importance. Economic evaluation of interventions to improve the management of diabetes have, in recent years, increasingly relied on modelling of long term outcomes and costs of disease [22]. Simulation models in diabetes typically project both life expectancy and the degree of morbidity associated with the disease. These estimates can assist in resource planning and can be used to inform economic evaluations, as they quantify potential savings that accrue when interventions reduce the risk of diabetes-related complications. Currently many diabetes simulation models use annual cycles to predict a profile of events over time [23,24]. The benefits of the present intervention are expected to be found in reduced health care and drug utilisation, and increased productivity (measured as reduced sickness absence and increased employment). More specifically, increased physical activity is expected to, among others, reduce the risk of cardiovascular diseases and improve health related quality of life.

### Aims

The first aim of the study is to examine whether an extended multi-professional PAR intervention, based on SDT, is effective in increasing and maintaining the self-reported physical activity level in patients with newly diagnosed type 2 diabetes or hypertension or both.

The second aim is to evaluate changes in a number of health-related variables such as blood pressure, plasma glucose, need of pharmacological therapy, health related quality of life, anxiety and depression, behavioral predictors, physical fitness, obesity, and activities of daily living.

The third aim is to perform an economic evaluation of the intervention from a societal perspective to allow for efficient use of societal resources.

## Methods/design

The study was initiated by Dalby health care centre and involves three primary care centres in Skåne, southern Sweden. Each primary care centre in Sweden has a population of listed patients; the 155 primary care centres in Skåne have between 3000–15500 listed patients and 10–40 employees, e.g. physicians, nurses, physiotherapists, occupational therapists, dieticians, and behavioural scientists.

In the present study, each of the three primary care centers constitutes one of three treatment groups: 1) Intervention group: extended PAR intervention based on a multi-professional team analysis of the study questionnaire and a 6-minute walk test, 2) Control group A: study questionnaire and physical activity prescription in usual care and 3) Control group B: treatment as usual (retrospective data collection). The two control primary care centres have similar socioeconomic and living environment profiles as the Intervention centre. At the health care centres in the Intervention group and Control group A, all patients with newly debuted type 2-diabetes or hypertension will be continuously asked to participate by their general practitioners (GPs).

The study is designed as a long-term intervention that will extend over several years with regular analyses of the patients’ self-reported physical activity levels, health-related variables and cost-effectiveness.

All parts of the study will be conducted according to the principles of the Helsinki Declaration. Written, informed consent will be obtained from all patients entering the Intervention group or Control group A before inclusion.

### The 5 A’s model

**Figure 3:**
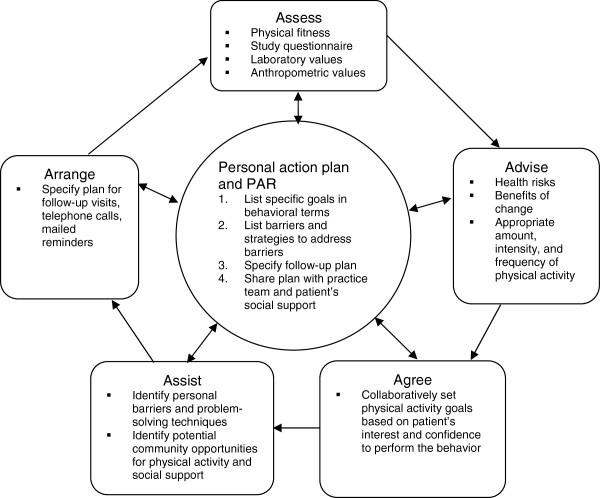
**The 5 A’s model.** Adapted for the extended PAR intervention from [25,26].

The study will be performed according to the 5 A’s model, which has been the basis for many health behavior change programs [25] and is especially effective for long-term interventions [26]. The model comprises the following 5 steps: Assess; Advice, Agree; Assist; and Arrange, which are continuously followed-up in relation to a personal action plan The contents of each step are presented in Figure 3.

The 5 A’s will, in our approach, involve the whole primary care team as well as the patients; different parts of the model will be performed by different team members and some parts may even involve family members or local public health and sports organizations.

### Intervention

**Figure 4:**
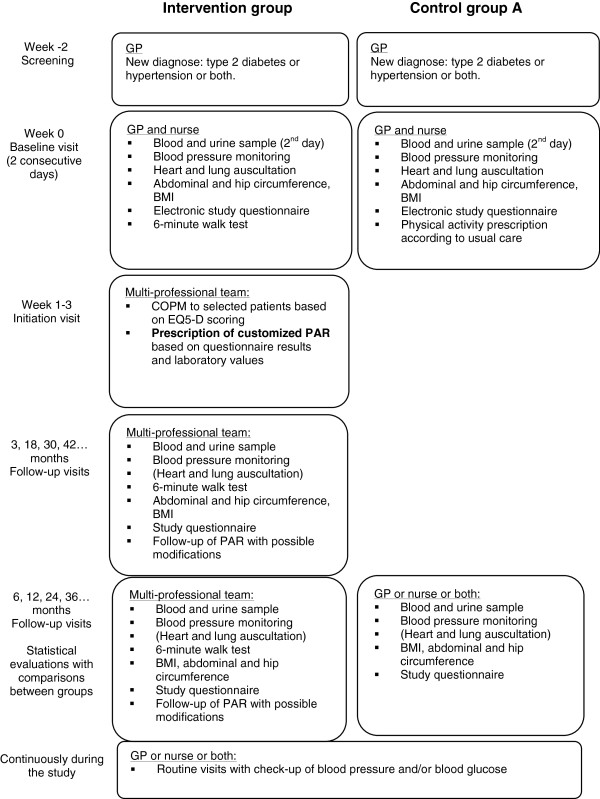
**Study flowchart.** Flowchart describing the study-specific patient visits. In addition to the study-related visits, the patients will be called to routine visits at the health care centers regularly for blood pressure and plasma glucose check-ups. The flowchart does not include Control group B, which will be analyzed retrospectively. Abbreviations: GP = general practitioner; PAR = physical activity referral.

A flowchart describing the time course of the study for the Intervention group and Control group A is presented in Figure 4. Below follows a short description of the intervention.

#### Intervention group

Eligible patients in the intervention group that consent to take part in the study will go through an extensive intervention program, in which a prescription of physical activity (PAR) will be the central part. At the *Baseline visit*, the patient meets his/her GP and a nurse. Values for blood pressure (in sitting position after 5 minutes rest; mean of two measurements, supplemented with 24 hour blood pressure monitoring), body mass index (BMI), and abdominal and hip circumference will be taken and a routine heart and lung auscultation and electrocardiography (ECG) will be performed. Furthermore, the patients will fill in an electronic study questionnaire regarding current health status, including variables such as level of physical activity; health behaviours; stress and anxiety; behavioural predictors; health related quality of life; sleeping habits; and activities of daily living ability. The patients will also perform a 6-minute walk test according to a separate, standardized protocol and guided by a physiotherapist. Fasting plasma glucose and other study specific blood samples will be taken in the morning within a few days.

These measures are in accordance with the first and part of the second step of the 5 A’s model (Figure 3).

After the baseline visit, the patients will be referred to an *Initiation visit* with one or more members of the multi-professional team comprising psychologist, physiotherapist, occupational therapist, physician and nurse as applicable. The initiation visit will take place within 3 weeks from the baseline visit.

The visit will be based on a team discussion regarding the results of the extended screening including the study questionnaire, the 6-minute walk test and baseline values. It will be structured according to step 2–5 in the 5 A’s model (advise, agree, assist and arrange; Figure 3) to provide the patients with a personal action plan based on individual goals and strategies to overcome personal barriers to physical activity. The initiation visit will be founded on the patients’ present level of motivation for exercise: amotivation, extrinsic motivation or intrinsic motivation, as described in the SDT model (Figure 1). To improve the patients’ autonomy, competence and relatedness regarding a more active lifestyle, the team uses the MI-technique throughout the conversation (Figure 2). It should be noted that all prescribers have attended at least one course in MI*.* The resulting action plan may include referrals to specialists (psychologist, physiotherapist and/or occupational therapist) for individual consultations and treatments to improve internal motivation and/or physical abilities. The discussion will result in a customized PAR, usually prescribed by a psychologist or physiotherapist from the team together with the patient. The PARs will be tailored to fit the patients’ physical and mental condition and motivation, and the recommendations in the Swedish report Physical activity in prevention and treatment of diseases, FYSS, as applicable [27].

#### Control group A

The patients in Control group A will go through the same physical examinations and study questionnaire as the patients in the Intervention group at the baseline visit. Apart from this, they will get treatment as usual including physical activity prescription as applicable. For Control group A, this is the only study-related visit during the initiation period. They will not attend an additional initiation visit, perform the 6-minute walk test or get a multi-professional team opinion on their treatment.

#### Follow-up visits

The patients in the Intervention group and Control group A will return to the clinics for follow-up of the study results regularly (Figure 4). During these follow-up visits, a physical examination, including monitoring of blood pressure and fasting plasma glucose and, if required, a heart- and lung auscultation will be performed. BMI, abdominal and hip circumference will be measured and the patients will fill in the same study questionnaire as before or a short version of the study questionnaire, including questions about their activity level. The patients in the Intervention group will also perform regular 6-minute walk tests.

The multi-professional team will, together with the patient, follow up adherence to the physical activity prescription, and modify it if necessary.

#### Routine visits

In addition to these study-related visits, the patients will, if necessary, be called to routine visits at the health care centers for blood pressure and plasma glucose check-ups regularly.

#### Control group B

The patient data in Control group B will be collected from the medical records of patients, diagnosed during the same time period as the other two groups, retrospectively. The same baseline data will be collected as in the other two groups, i.e. time to initiating pharmacological treatment, number of medications and laboratory as well as clinical data. The data will be collected on a population basis.

### Inclusion and exclusion criteria

The main inclusion criteria are newly diagnosed type 2 diabetes and/or hypertension. Consecutive primary care patients with suspected type 2 diabetes or hypertension will be screened for inclusion. The diagnose criterion for type 2 diabetes in both the Intervention and the Control groups is defined as a 2 x fasting plasma glucose (fP-glu) ≥ 7.0 mmol/l according to WHO guidelines [28]. The diagnose criterion for hypertension is defined as a resting systolic blood pressure ≥ 140 mmHg or a diastolic blood pressure ≥ 90 mmHg. Hypertension is confirmed by a 24-hour ambulatory blood pressure monitoring: a systolic blood pressure ≥ 135 mmHg and/or a diastolic blood pressure ≥ 85 mmHg is considered confirmatory of hypertension since ambulatory blood pressure is usually a few mmHg lower than office blood pressure [5]. If the resting blood pressure exceeds 160/100 mmHg at two consecutive measurements within one week, the patient will be prescribed pharmacological treatment in addition to physical activity. Patients with a history of type 2 diabetes or hypertension that get diagnosed by the other diagnose will be included in the study as a subgroup, in which a reduction of current medication is one endpoint. On the other hand, patients with a history of type 1 diabetes will be excluded from the study. The overall inclusion and exclusion criteria of the study are presented in Table 1.

**Table 1:** Overall inclusion and exclusion criteria

**A Overall inclusion criteria**	
1. Newly diagnosed type 2 diabetes (a) or hypertension (b) or both	
a) Type 2 diabetes	b) Hypertension
2 x fasting plasma glucose (fP-glu) ≥ 7.0mmol/l	A systolic blood pressure ≥ 140mmHg and/or a diastolic bloodpressure ≥ 90 mmHg, which isconfirmed by a systolic bloodpressure ≥ 135 mmHg or a-diastolic blood pressure ≥ 85mmHg during a 24-hourambulatory monitoring
2. Male and female patients ≥ 18 years	
**B Overall exclusion criteria**	
1. < 18 years	
2. History of type 1 diabetes	
3. Blood pressure > 180/105 mmHg [29]	
3. Known pregnancy	
4. Cognitive disabilities that are likely to limit adherence to intervention	
5. Cardio- or cerebrovascular event that required hospitalization within the past 3 months	
6. Current participation in other clinical study	
7. Severe mental disorder or substance abuse	

### Outcome measures

#### Primary outcome

Level of self-reported physical activity (as assessed with the International Physical Activity Questionnaire (IPAQ) and expressed as MET-minutes).

#### Secondary outcomes

(1) Need of pharmacological therapy for hypertension or type 2 diabetes;

(2) Blood pressure/plasma glucose;

(3) Physical fitness and anthropometric variables;

(4) Mental health (anxiety, depression, sleep, stress);

(5) Health related quality of life; and

(6) Cost-effectiveness of intervention.

### Assessment of physical activity level (primary outcome)

Individual physical activity levels will be assessed with the International Physical Activity Questionnaire (IPAQ), a validated instrument for assessing physical activity [30], in the Intervention group and Control group A. IPAQ comprises 4 simple questions on physical activity, which will be included in the study questionnaire. The IPAQ results will be expressed as MET-minutes per week. A metabolic equivalent, or MET, is a unit that describes the energy expenditure of a specific activity. A MET is the ratio of the rate of energy expended during a specific activity to the resting metabolic rate. 1 MET is equivalent to the resting metabolic rate, while a 2 MET-activity requires two times the metabolic energy expenditure of sitting quietly [31]. If a person does a 2 MET-activity for 30 minutes, he or she has done 2 × 30 = 60 MET-minutes of physical activity.

### Assessment of physical fitness and anthropometric variables (secondary outcome)

Physical fitness will be estimated with the 6-minute walk test in the Intervention group only. The 6-minute walk test measures the distance that a patient can walk on a flat, hard surface in 6 minutes, usually on a short indoor track. This test is useful as a complement to the subjective assessment in the study questionnaire since it evaluates the integrated responses of all body systems that are involved in physical activity including the pulmonary and cardiovascular systems, systemic and peripheral circulation, blood, neuromuscular units and muscle metabolism [32]. In the present study, the 6-minute walk test will be used to evaluate changes in physical fitness expressed as percentage of the individual result at baseline. The patients will perform a “test-walk” for 1 minute before the 6-minute walk test and the test will be performed according to a separate, standardized protocol [32].

Anthropometric measurements will include weight, length, body mass index and waist-to-hip ratio.

### Assessment of behavioural predictors, mental health and health related quality of life (secondary outcome)

With the study questionnaire, psychological variables will be collected to assess a) behavioural predictors, b) mental health and c) health related quality of life. These variables include: a) self-perceived health, self efficacy, self-determination; b) anxiety and depression, stress, panic-related physical sensations, and sleeping quality; c) health related quality of life (Table 2). Almost all these items will be evaluated with questions from already validated questionnaires, which will be included in the study questionnaire. If a person in Control group A gets results that indicates severe depression or anxiety, this person will be offered a medical appointment.

**Table 2:** Study questionnaire variables with references

**Variable**	**Reference**
Demographic and socioeconomic characteristics and other general questions concerning background and health related issues like general health, current medications, symptoms, pain, stress, and sleeping quality.	*The Public Health Survey* in Skåne, Sweden and questions from the Swedish Annual Level of Living Survey (SALLS).
Health related quality of life	EQ-5D (http://www.euroqol.org/)
Lifestyle related habits - general questions related to the individuals: physical activity, healthy eating, tobacco and alcohol usage	The National Board of Health and Welfares Questionnaire.
Physical activity level	International Physical Activity Questionnaire (IPAQ) – short version [30].
Stages of change (readiness for change to be more physically active)	Used in previous study [9]
Physical activity history and dog ownership and dog-walking	Study specific question
Self-efficacy	General Self-Efficacy Scale (http://www.ralfschwarzer.de/)
Self-determination	Perceived Competence Scale (PCS), Health Care Climate Questionnaire (HCCQ), Treatment Self-Regulation Questionnaire (TSRQ) from the Health-Care, Self-Determination Theory Packet (http://www.psych.rochester.edu/SDT/measures/hc_description.php)
Anxiety and depression	Hospital Anxiety and Depression (HAD) Scale [33]
Anxiety related physical sensations	Anxiety Sensitivity Index (ASI) [34]
Activities of daily living (ADL) ability	Canadian Occupational Performance Measure (COPM) [35]
- only patients that score 2–3 p in question A, B, or C in EQ-5D	
Non-adherence and reasons for non-adherence to PARs intervention	Used in previous study [36]

### Assessment of cost effectiveness of intervention (secondary outcome)

The Intervention group will be compared with the two control groups in order to perform cost-effectiveness analysis (CEA) or cost-utility analysis (CUA) or both. All the costs related to the intervention such as training of the personnel and participants’ time and travel costs will be counted. Productivity losses (including informal care) and benefits will be counted using appropriate methods such as the friction cost and the human capital approach [37,38]. The project in general and the study questionnaire in particular, will be designed to effectively capture the benefits of the intervention, for example, the EQ-5D instrument will be used to examine changes in health related quality of life (http://www.euroqol.org). The intermediate outcomes of the intervention (e.g. reduction of cardiovascular risk factors) will be extrapolated by economic modelling (either Decision tree or Markov model) to predict the long term health related benefits [39,40]. Generating estimates of acute and long-term costs associated with the management of diabetes requires information on the annual average health care costs with a wide spectrum of complications. The most significant costs associated with many complications are likely to be those arising from inpatient hospital episodes. These costs have been estimated for selected countries, for example Sweden [41].

### Electronic study questionnaire

The study questionnaire will be filled in directly on a computer at the health care centre under supervision of a health care professional. All data are then submitted to a patient database, which automatically provides instant feed-back on the patients’ health status. The study questionnaire comprises questions from validated question batteries, questions from the regional population survey, and some questions specifically designed for the study (Table 2), in order to thoroughly evaluate the patients’ health status as well as the study methodology. A short-version of the questionnaire will be used at some of the follow-up visits (see Figure 4).

### Laboratory values

A study specific blood sample, drawn after an overnight fast, and a morning urine sample will be taken at all study-specific visits to measure lipid profile, plasma glucose, HbA1c, haemoglobin, electrolyte status, thyroid status, high-sensitive C-reactive protein and urinary albumin-to-creatinine ratio. The samples will be analyzed at the Department of Clinical Chemistry, Skåne University Hospital. One frozen blood sample will be collected for long-term storage for potential future analysis.

### End of study

The study, including regular data collection, is designed as a longitudinal intervention that will last over years. The patients will be invited to participate in the intervention until further notice or until they choose to finish their participation themselves (not equivalent to non-adherence to intervention).

The intervention may be extended to evaluate other endpoints than the ones described in the present paper. In that case, ethical approval will be applied for separately.

### Adverse events

At the follow-up visits, the patients will be asked if they have experienced any adverse events since the last visit. All adverse events will be reported, independent of possible relation to the intervention.

### Statistical analysis and power calculation

The first statistical analyses and compilation of results will be performed 18 months after inclusion of the first patient or when the Intervention group and Control group A have included and followed up 60 and 30 eligible patients respectively for at least six months. At that point, data will also be collected retrospectively in control group B. Reasons for non-attendance in the study will be analyzed as well as reasons for non-adherence to the intervention. Allocation will be concealed, and study data blinded, for the bio-statisticians and researchers who will perform the analyses.

The sample size is based on an assumed statistical power of 80%, a significance level of 5%, a difference of 200 MET-minutes between the groups, a standard deviation of 300 MET-minutes and a worst-case loss to follow-up of 30%.

The secondary outcomes blood pressure, plasma glucose, need of pharmacological therapy, and cost-effectiveness will be compared between all three study groups.

### Approval and registration

The study was approved by Lund regional ethical review board, registration number 2010/330, and registered at ClinicalTrials.gov, registration number NCT01187576.

## Discussion

The targeted patients – individuals with newly diagnosed type 2 diabetes or hypertension – belong to a patient category that is indeed supposed to benefit from increased physical activity in terms of improved health variables, decreased risk of complications [27], and possibly also a decreased need of pharmacological therapy [42]. In the present study we combine a patient-centered lifestyle modification by a multi-professional team; a theory-based approach for self-regulation and behavior change; a feedback system for immediate response to treatment and adherence to treatment; and determination of cost-effectiveness of the intervention. This is in agreement with current guidelines for the prevention and management of the targeted diseases [5,6], and may increase the efficiency of treatment and evaluation of intervention.

The intervention intends to increase the patients’ internal motivation for physical activity by following the principles of SDT and MI, and thereby improve their adherence to PAR. The ultimate goal is to decrease cardiovascular complications, improve quality of life and work ability and decrease the need of medication. Our hypothesis is that the proposed PAR intervention is a more effective way to support patients in achieving and maintaining a beneficial level of physical activity than treatment as usual. We also believe that this structured intervention can be cost effective if it leads to a better health status and, subsequently, a less need of medical care among these patients.

The reason for using two control groups is that we believe there is a risk that the patients in Control group A may get an increased awareness of physical activity and subsequent alteration of habits, just by participating and filling in the questionnaires. Therefore we are also making a retrospective analysis at a third centre.

The study has some limitations. One possible limitation is the uncertain generalizability of the findings if the results of the intervention are related to individual qualities of the persons delivering the intervention, such as a special expertise and enthusiasm. Other possible limitations of lifestyle intervention studies in general and physical activity interventions in particular are the difficulties to determine relevant differences between the groups for the power calculation. The assumed difference of 200 MET-minutes is an ambitious goal and a balance between assumed effect of intervention, time to follow-up, and assumed number of patients. The primary outcome may not turn out significant at the first analysis (after one year). We believe, however, that 200 MET-minutes is a reachable difference and that the difference between the groups will increase over time.

If the extended PAR intervention proves to be successful, the model may be adopted by other primary care centers as well. Our goal is to find an attractive, user-friendly and cost effective PAR scheme, which may be translated to a broad range of patient groups that benefit from increased physical activity.

## Abbreviations

ADL: Activities of daily living; BMI: Body mass index; CA: Control group A; CB: Control group B; ECG: Electro cardiography; fP-glu: Fasting plasma glucose; IG: Intervention group; IPAQ: International physical activity questionnaire; MET: Metabolic equivalent; MI: Motivational interviewing; PAR: Physical activity referral; SDT: Self-determination theory.

## Competing interests

The authors declare that they have no competing interests.

## Authors’ contributions

RE (MD, PhD and head of Dalby Health Care Centre) introduced the idea and initiated this study. Research Coordinator and Biologist ES (PhD), Research Coordinator Public Health specialist ML (PhD), Psychologist CB, Public Health Nutritionist DA (PhD) and the Family Physicians KS (MD, PhD), SC (MD, PhD), and RE formulated the aim of the study and set up the study logistics. ES drafted the present study protocol. ML coordinated and drafted the study questionnaire. Health Economist UG (PhD) introduced the health economic aspects of the project. All authors contributed to developing the project and all authors read, commented and approved the final version of the manuscript.

## Pre-publication history

The pre-publication history for this paper can be accessed here:

http://www.biomedcentral.com/1472-6963/12/173/prepub
